# Broad and long-lasting immune protection against various Chikungunya genotypes demonstrated by participants in a cross-sectional study in a Cambodian rural community

**DOI:** 10.1038/s41426-017-0010-0

**Published:** 2018-02-07

**Authors:** Heidi Auerswald, Camille Boussioux, Saraden In, Sokthearom Mao, Sivuth Ong, Rekol Huy, Rithea Leang, Malen Chan, Veasna Duong, Sowath Ly, Arnaud Tarantola, Philippe Dussart

**Affiliations:** 1 Virology Unit, Institut Pasteur du Cambodge, Institut Pasteur International Network, PO Box 983, Phnom Penh, Cambodia; 2 National Center for Parasitology, Entomology, and Malaria Control, Phnom Penh, Cambodia; 3 Epidemiology and Public Health Unit, Institut Pasteur du Cambodge, Institut Pasteur International Network, PO Box 983, Phnom Penh, Cambodia; 4 Unité d’Epidémiologie, Institut Pasteur de Nouvelle-Calédonie, Institut Pasteur International Network, Noumea, New Caledonia

## Abstract

Chikungunya virus (CHIKV) is an alphavirus circulating worldwide. Its presence in Asia has been reported since the 1950s, constituting the Asian genotype. Since 2005, strains from the Eastern, Central, and Southern African (ECSA) genotype have caused several outbreaks across Asia. Viruses from the ECSA genotype were also detected in Cambodia in late 2011 and led to an outbreak in a rural community in 2012. A former investigation from 2012 found a higher risk of infection in people younger than 40 years, suggesting a pre-existing herd immunity in the older Cambodian population due to infection with an Asian genotype. In 2016, we collected serum from equivalent numbers of individuals born before 1975 and born after 1980 that were also part of the 2012 study. We analyzed the 154 serum samples from 2016 for neutralization against the Cambodian ECSA isolate and three strains belonging to the Asian genotype. This experiment revealed that 22.5% (18/80) of the younger study participants had no CHIKV antibodies, whereas 5.4% (4/74) of the older population remained naive. Study participants infected during the ECSA outbreak had twofold neutralizing titers against the ECSA and the most ancient Asian genotype virus (Thailand 1958) compared to the other two Asian genotype viruses. The neutralization data also support the older population’s exposure to an Asian genotype virus during the 1960s. The observed cross-reactivity confirms that the investigated CHIKV strains belong to a single serotype despite the emergence of novel ECSA genotype viruses and supports the importance of the development of a Chikungunya vaccine.

## Introduction

Chikungunya is a mosquito-borne viral disease transmitted by *Aedes* (*Ae*.) mosquitoes. The chikungunya virus (CHIKV) is enveloped and belongs to the family *Togaviridae*, genus *Alphavirus* and further to the Semliki Forest virus antigenic complex that also contains the O’nyong-nyong, Mayaro, and Ross River viruses. Its single-stranded, positive-sense RNA genome (~12 kb) encodes four nonstructural proteins (nsP1 to nsP4) and five structural proteins (Capsid, E3, E2, 6k, and E1). Three distinct genotypes have been defined based on E1 envelope glycoprotein sequences^1–3^. CHIKV has emerged and caused a series of outbreaks in recent decades, mainly transmitted by *Ae. aegypti* mosquitoes. It was first described in 1952 in Tanzania^4, 5^ and was later identified as a virus belonging to the consequently named Eastern, Central, and Southern Africa (ECSA) genotype^6^. CHIKV spread in the 1950s to Asia, where viruses of the Asian genotype have circulated since. In 2004, the ECSA genotype spread from Kenya^7^ eastward across several islands in the Indian Ocean^8–10^ to India^11^ and finally emerged in Southeast Asia^12–16^. It acquired mutations on the way^3, 17^, leading to enhanced replication in *Ae. albopictus* mosquitoes^18–20^. Viruses with these adaptive mutations were grouped into the ECSA Indian Ocean Lineage (IOL). Furthermore, CHIKV IOL viruses were imported to Italy^21^ and France^22^ due to the wide distribution of *Ae. albopictus*, causing the first—albeit limited—CHIKV outbreaks outside the tropical setting^23^.

The newly described transmissibility of CHIKV by this vector species and the fact that the outbreaks affected regions with nearly CHIKV-naive populations were the main drivers for explosive epidemics in the Indian Ocean and Asia, with high attack rates and morbidity. The same was true when CHIKV re-emerged in Cambodia in 2011^15^, five decades after it was detected in 1961^24^. The latest outbreak, caused by the IOL strain in 2012, was studied in detail in the rural community of Trapeang Roka, Kampong Speu province, Cambodia^25, 26^. In contrast to other studies on recent IOL outbreaks, which showed associations between higher risk for infection and older age^27–29^, this field investigation in Cambodia found a lower risk for CHIKV infection in people born before 1975^26^. This finding led to the hypothesis that those born before 1975 were pre-immune to IOL CHIKV in 2012 due to exposure to CHIKV of the Asian genotype in the 1960s or early 1970s. We sought to document the relationship between age and protection against different CHIKV strains using a neutralization assay.

Long-lasting immunity against CHIKV re-infection is known from epidemiological observations^26, 30^, as well as vaccine trials in animal models^31–33^, and maybe lifelong. The presence of neutralizing antibodies is associated with protection from disease, as shown in diverse mouse models^34, 35^, and is suggested by prospective data from the Philippines^36^. Neutralizing antibodies mainly target the CHIKV envelope glycoproteins E1 and E2^37, 38^. As E2 contains putative-binding sites to an unknown receptor and is most exposed on the virion surface, it also contains the epitopes for most of the neutralizing antibodies that block the endocytose-mediated entry of viral particles^39^. Another virus neutralization mechanism blocks the fusion of the viral envelope protein with the membrane of the host cell’s endosome^40^, preventing the release of the viral nucleocapsid into the cell. Antibodies that use this mechanism target the fusion peptide located in the E1 protein^41^. Despite the co-circulation of three genotypes differing mainly in their envelope sequences, only one CHIKV serotype is described. This explains why CHIKV epidemics are explosive in non-immune populations but then vanish for decades^6^.

This study aimed to investigate the relationship between age and protection against different CHIKV strains using a neutralization assay, as a previous observation found evidence for pre-existing immunity in people born before 1975^26^. We analyzed serum samples, collected 5 years after the 2011 emergence, from individuals born before 1975 and after 1980. We determined the neutralizing antibody titers in people born before 1975 and after 1980 against the Cambodian IOL strain isolated in 2011 and against three Asian genotype strains isolated in different decades.

## Materials and methods

### Study design and setting

In March 2012, several CHIKV infections were reported among village residents of Trapeang Roka in Kampong Speu Province, Cambodia after CHIKV re-emerged through the country and spread in 2011^15^. A subsequent field investigation was conducted among village residents by the Cambodian Communicable Disease Control Department, National Malaria Center, Institut Pasteur du Cambodge (IPC), local health centers, and village authorities to document the outbreak and gather information for response planning and control efforts^25^. This first serosurvey was a point-prevalence cross-sectional study, conducted on a single day, 3–4 weeks after the detection of the index cases. Approximately 60% of the village population was enrolled in this survey, and CHIKV affected families throughout the village. In total, 44.7% (190/425) of the population tested had evidence of infection by CHIKV, which affected all age groups. Only 2 patients of 190 remained febrile and tested positive by CHIKV RT-PCR. The epidemic curve suggested that CHIKV lasted ~3 weeks and was ending when this serosurvey was conducted in late March 2012^25^. Finally, pre-existing immunity against CHIKV was not explored during this first survey,as neither CHIKV IgG nor CHIKV neutralization assays were performed. Our study explored the hypothesis of pre-existing immunity in the older population that was indicated by this Trapeang Roka field investigation^26^. To achieve this aim, a second point-prevalence cross-sectional study was conducted in October 2016. The inclusion criteria were as follows: inhabitants of Trapeang Roka village who participated in the 2012 outbreak investigation, who were tested for anti-CHIKV IgM during that investigation and for whom informed consent to participate in the present study was obtained. Participants were recruited to include equivalent numbers of people for both age groups (born before 1975 and after 1980). A volume of 5 ml whole blood was collected in plain tubes for CHIKV serological diagnosis. Specimens were kept at 4 °C after sampling and transported to the IPC laboratory, where the serum was separated immediately by centrifugation and was frozen at −80 °C until further analysis.

### Ethics statement

This study was approved by the Cambodian National Ethics Committee for Human Research (approval #261NECHR/2016). All human samples were collected after obtaining informed consent from the patients, parents, or guardians.

### Cells

VeroE6 cells were used for the detection of neutralizing antibodies via the foci reduction neutralization test (FRNT). These cells were cultivated in Dulbecco’s modified Eagle medium (DMEM; Sigma-Aldrich, Steinheim, Germany) supplemented with 10% fetal bovine serum (FBS; Gibco, Gaithersburg, MD, USA) and 100 U/ml penicillin-streptomycin (Gibco) at 37 °C in a 5% CO_2_ atmosphere. CHIKV isolates were grown in C6/36 *Ae. albopictus* cells and harvested from the supernatant. The mosquito cells were cultured in Leibovitz-15 medium (Sigma-Aldrich) supplemented with 10% FBS, 1% l-glutamine (Gibco), 10% tryptose-phosphate (Gibco), and 100 U/ml penicillin-streptomycin at 28 °C.

### Viruses

We used the following CHIKV strains in this study (Supplement Table S1): two Thai CHIKV strains, TH 35 (GenBank accession no. HM045810), and TH 1455-75 (GenBank accession no. AF192898), belonging to the Asian genotype and isolated from humans in Thailand in 1958 and 1975, respectively. These viruses were obtained as lyophilized stocks from the World Reference Center for Emerging Viruses and Arboviruses at the University of Texas Medical Branch (Galveston, TX, USA). The other Asian genotype virus strain used, CHIKV NC-2011-568 (GenBank accession no. HE806461), was isolated from a patient in New Caledonia in 2011 and was kindly provided by Dr Myrielle Dupont-Rouzeyrol, URE Dengue et Arboviroses, Institut Pasteur de Nouvelle-Calédonie. Finally, the CHIKV Cambodian strain, belonging to the ECSA genotype, was isolated from a human during the re-emergence of CHIKV in Cambodia at the end of 2011 (GenBank accession no. JQ861253).

### IgM antibody-capture enzyme-linked immunosorbent assay

The dried blood spots obtained during the outbreak investigation were tested in 2012 for CHIKV IgM with an in-house IgM antibody-capture enzyme-linked immunosorbent assay (MAC-ELISA) using antigen originating from the CHIKV Ross C 347 strain^15, 42^. A result was considered positive for CHIKV when the optical density (OD) was higher than 0.1 (threshold determined by measuring the OD of CHIKV-negative human serum). The presence of IgM antibodies in these 2012 samples confirmed CHIKV infection during the 2012 outbreak^26^.

### Hemagglutination inhibition assay

The presence of antibodies in the serum samples collected in 2016 was tested by hemagglutinin inactivation assay (HIA) using antigen originating from the CHIKV Ross C 347 strain. The assay followed the protocol described by Clark and Casals adapted to 96-well microtiter plates^43^.

### Foci reduction neutralization test

The FRNT micro-neutralization assay using different CHIKV strains from Southeast Asia and the Pacific region (Supplement Table S1) determined the level of neutralizing antibodies. The serum samples collected in 2016 were thus analyzed by FRNT using VeroE6 cells seeded in 96-well plates. Heat-treated sera were serial diluted (from 1:10 to 1:5120) and mixed with an equal volume of virus (800–1200 ffu/ml). All sera were tested in triplicate. Virus-serum mixtures were incubated for 1 h at 37 °C and then used for inoculation of VeroE6 monolayers. After 1 h of incubation at 37 °C the virus-serum mixtures were replaced by a semi-solid overlay containing 1.6% carboxymethyl cellulose (Sigma-Aldrich) in DMEM medium supplemented with 3% FBS. The plates were incubated at 37 °C in a5% CO_2_ atmosphere and stained 18–24 h after infection. Cells were fixed with 4% paraformaldehyde (Sigma-Aldrich) in phosphate-buffered saline (PBS) for 30 min. The plates were then incubated sequentially with 0.5% Triton X-100 (Sigma-Aldrich) in PBS for 20 min and with 10% FBS in PBS, polyclonal anti-CHIKV mouse hyper immune ascites fluids (IPC, Cambodia), and anti-mouse IgG antibody conjugated to horseradish peroxidase (Bio-Rad, Marnes La Coquette, France) for 1 h each. Finally, the infected cells were visualized with TrueBlue peroxidase substrate (KPL, Gaithersburg, MD, USA). The neutralization titer demonstrated the reciprocal serum dilution that induced a 90% reduction of the foci number (FRNT_90_) compared to the controls (flavi virus-negative control serum and virus dilution without added serum) and was calculated via log probit regression analysis (SPSS for Windows, Version 16.0, SPSS Inc., Chicago, IL, USA). FRNT_90_ titers below 20 were considered negative.

#### Statistical analysis

Statistical analyses were performed using GraphPad Prism for Windows, version 7.02 (GraphPad Software, Inc., La Jolla, CA, USA). Comparative analyses for HIA titers between two independent groups were carried out using the unpaired *t* test. Additionally, comparative analyses of the size of certain subgroups were performed with the two-tailed *χ*^2^ test with Yates correction. To compare positive FRNT_90_ titers between different groups (age group: born before 1975 versus after 1980; IgM status: IgM positive versus IgM negative), a one-way ANOVA test was used followed by Tukey’s multiple comparison testing. The analysis of correlation between HIA and FRNT assays performed with different CHIKV strains with regard to antibody titers was undertaken using Spearman’s correlation test. A significance level of 0.05 was used for all tests.

## Results

**Fig. 1:**
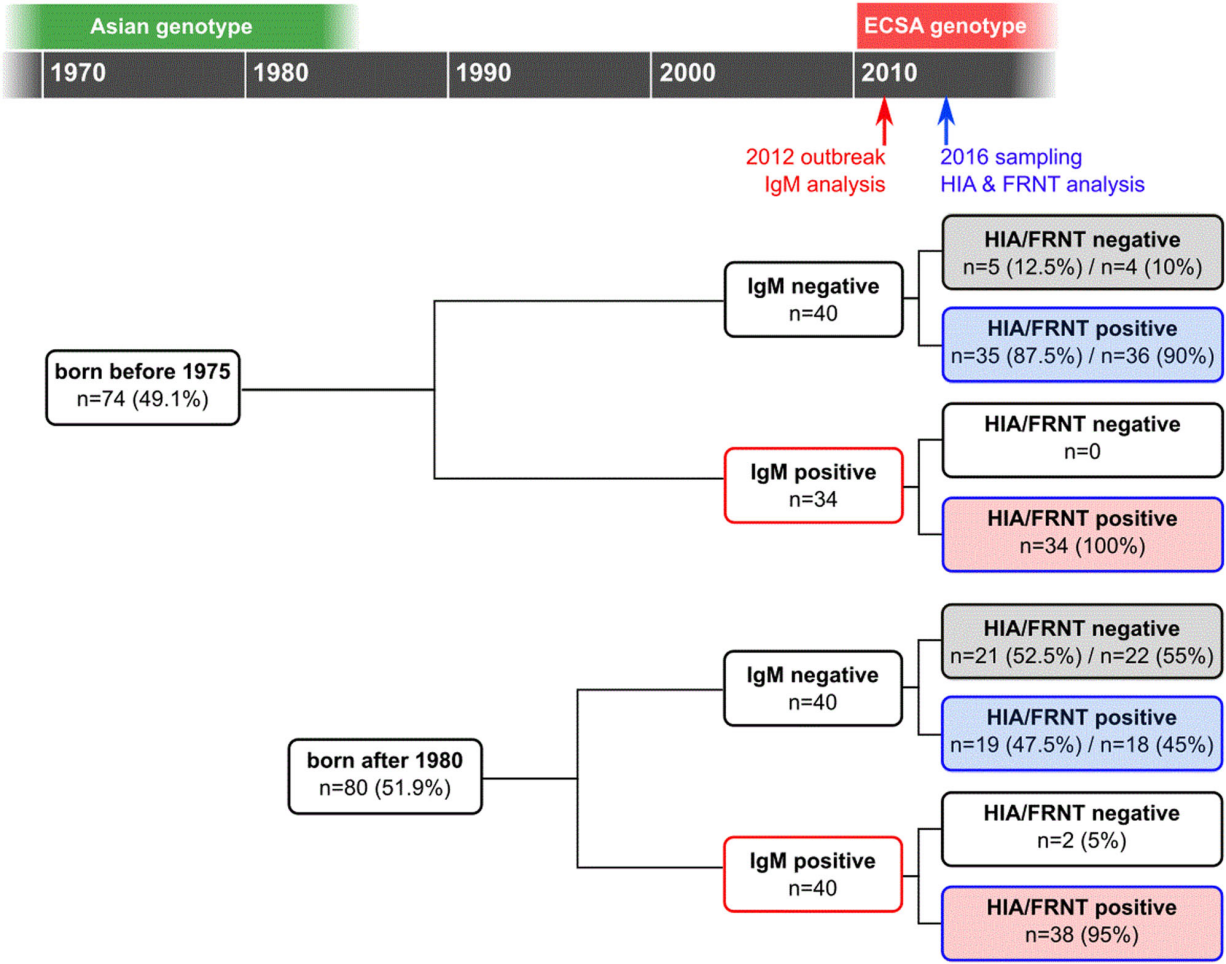
Study design and outcome. The recruitment included a comparable number of individuals born before 1975 (*n* = 74) and after 1980 (*n* = 80), as well as numbers of infections (IgM positive) confirmed in 2012. Based on the IgM status from 2012 and the antibody status in 2016 (investigated by HIA and FRNT), the study participants could be classified as individuals who remained naive in 2016 (gray shaded boxes), individuals infected during the outbreak in 2012 (red shaded boxes), and study participants with CHIKV immunity acquired at an unknown time point (blue shaded boxes). All criteria allowed for a separate analysis of subgroups, designated subgroup 1 based on the age of the study participants, subgroup 2 based on the IgM status in 2012, and subgroup 3 including both age and 2012 IgM status

The study was conducted in October 2016 in the rural community of Trapeang Roka and included 154 participants who had been tested for IgM shortly after the 2012 CHIKV IOL outbreak: 74 participants born before 1975 and 80 participants born after 1980 (Fig. 1). The IgM status in 2012, as well as the HIA and FRNT_90_ status in 2016, of the study participants, stratified by age group (born before 1975 or after 1980), is shown in Table 1. The numbers of people who tested IgM negative and IgM positive in 2012 are comparable: 54% (40/74) IgM negative in the group born before 1975 versus 50% (40/80) IgM negative in the group born after 1980 (*p* = 0.73). The FRNT_90_ titers were compared by stratifying study participants by age (subgroup 1), their IgM status in 2012 (subgroup 2), or both criteria (subgroup 3).

**Table 1:** Results of the three chikungunya serological assays used (MAC-ELISA, HIA, and FRNT) among the study participants (*n* = 154)

	Born before 1975		Born after 1980		Comparison of age groups	Total	
	*n*	(%)	*n*	(%)	*p* value^a^	*n*	(%)
MAC-ELISA in 2012							
Negative	40	(54)	40	(50)	0.73	80	(52)
Positive	34	(46)	40	(50)		74	(48)
HIA in 2016							
Negative	5	(7)	23	(29)	0.0009	28	(18)
Positive	69	(93)	57	(71)		126	(82)
FRNT in 2016							
Negative	4	(5)	24	(30)	0.0002	27	(17.5)
Positive	70	(95.5)	56	(70)		127	(82.5)
MAC-ELISA in 2012 & HIA in 2016							
Both negative	5	(7)	21	(26)	0.0012	26	(17)
Both positive	34	(50)	38	(47.5)		72	(47)
Discrepant	35	(47)	21	(26)		56	(36)
MAC-ELISA in 2012 & FRNT in 2016							
Both negative	4	(5)	22	(27.5)	0.0002	26	(17)
Both positive	34	(46)	38	(47.5)		72	(47)
Discrepant	36	(48)	20	(25)		56	(36)
Total	74	(48)	80	(52)	—	154	(100)

*MAC-ELISA* IgM antibody-capture ELISA, *HIA* hemagglutination inhibition assay, *FRNT* foci reduction neutralization test

^a^Two-tailed *χ*^2^ test with Yates correction.

### Correlation of HIA and FRNT assay

The serum samples collected in 2016 were tested for CHIKV antibodies by HIA using the CHIKV Ross C 347 strain and by FRNT using three Asian genotype viruses and one Cambodian virus isolated at the end of 2011 that falls into the ECSA IOL genotype (Supplement Table S1). The HIA and FRNT antibody titers showed a strong positive correlation, with Spearman correlation coefficients ranging from 0.8 to 0.9 (Supplement Table S2), confirming the FRNT_90_ results by HIA. Two (1.30%) of the 154 analyzed sera had discrepant results for HIA and FRNT (Fig. 1): one study participant born before 1975 had an HIA titer below the cut-off of 20 and neutralizing antibodies against the CHIKV isolated in Thailand in 1954 (FRNT_90_ = 821) but no neutralizing antibodies against the other CHIKV strains. The other individual with discrepant results was born after 1980, showed an HIA titer just above the cut-off of 20, and was negative in the FRNT against all four CHIKV strains.The FRNT results for the different CHIKV strains also correlate positively (all *r* values >0.8; Supplement Table S2), confirming the known cross-reactivity between the CHIKV genotypes.

### Subgroup 1: Antibody levels in the different age groups

A comparison of study participants with positive detection for CHIKV antibodies in HIA and/or FRNT and individuals negative in these assays revealed significant differences (Table 1). Antibody detection by HIA revealed only five (6.8%) individuals in the study group born before 1975 without detectable antibodies, whereas in the group of people born after 1980, 23 (28.8%) individuals were negative with the HIA (*p* = 0.0009). This difference was confirmed by the FRNT results: four (5.4%) individuals born before 1975 had no neutralizing antibodies, whereas 24 (30%) study participants born after 1980 were still naive for CHIKV antibodies (*p* = 0.0002).

**Fig. 2:**
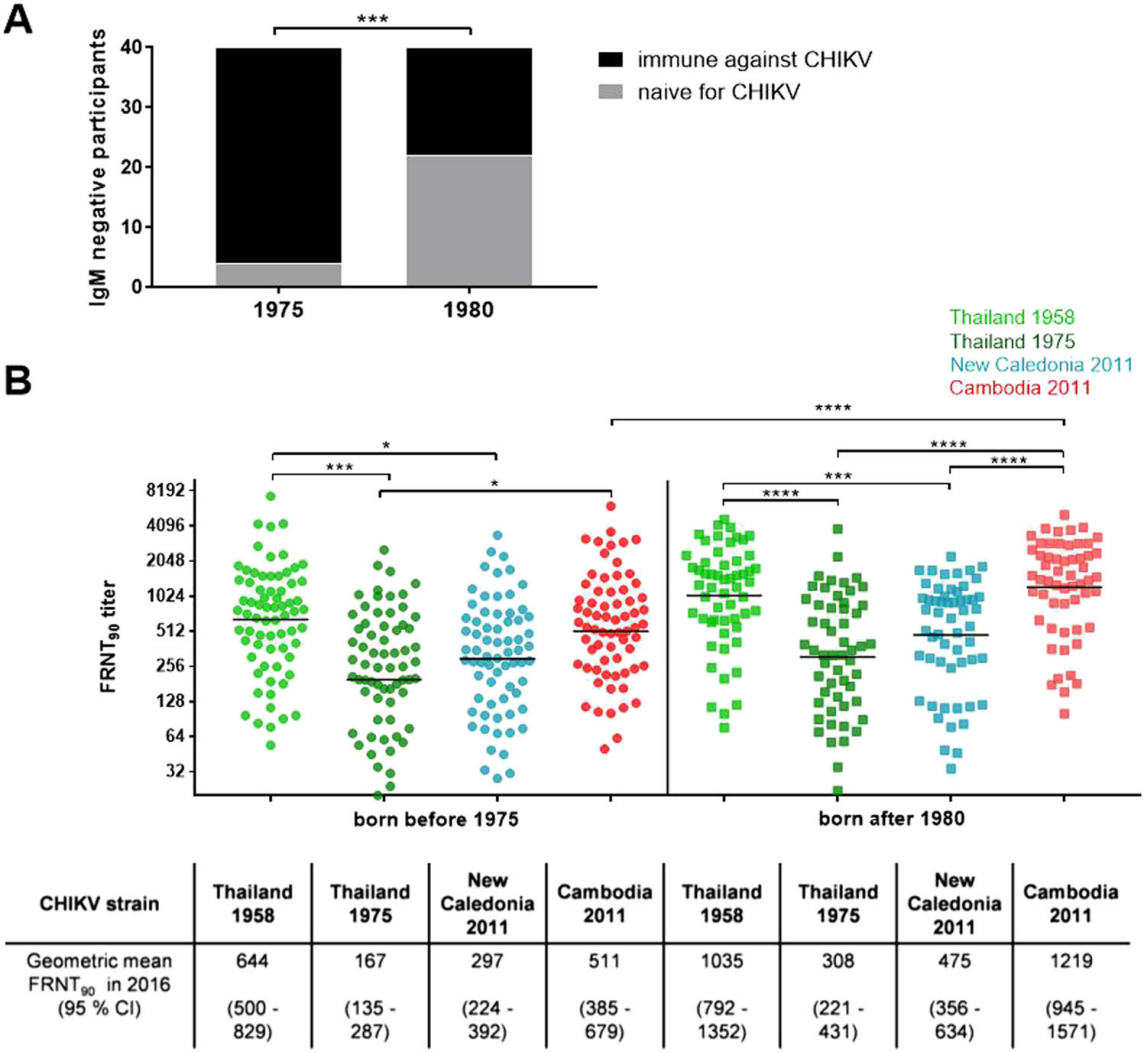
Subgroup 1: Influence of age on antibody levels. **a** Study participants who were not infected in 2012 (IgM negative) and were antibody positive in 2016 (positive FRNT_90_ titer; black bars) as well as individuals remaining naive in 2016 (negative FRNT_90_ titer; gray bars), stratified by age. Asterisks indicate statistically significant differences in the proportions of participants (***p < 0.001; *χ*^2^ test). **b** Individual FRNT_90_ titers (with geometric mean) of study participants with neutralizing antibodies (*n* = 126), stratified by age group (circles: born before 1975, *n* = 70; squares: born after 1980, *n* = 56). FRNT_90_ titers against the three Asian genotype strains from Thailand (TH 35: light green; TH 1455-75: dark green) and New Caledonia (NC-2011-568: blue) as well as the Cambodian ECSA IOL strain from 2011 (V1024306_KH11_PVH: red). Asterisks indicate statistically significant differences in mean FRNT_90_ titers between the distinct virus strains (*p < 0.05; ***p < 0.001; ****p < 0.0001; one-way ANOVA, Tukey’s multiple comparison test)

We compared the antibody titers measured by HIA and FRNT in the older (born before 1975; *n* = 74) and younger (born after 1980; *n* = 80) participants in our study groups (Fig. 1). The HIA titers were similar, with mean values of 320 and 431 for the older and younger study participants, respectively (*p* = 0.166; Table 2). The analysis of neutralizing antibodies in individuals who were not infected in 2012 revealed a significant difference between the age groups (Fig. 2a). We identified 22/40 (55%) of the younger study participants as immunologically naive in 2016, whereas only 4/40 (10%) of the older individuals had no detectable CHIKV-neutralizing antibodies (*p* < 0.0001), showing notably higher levels of CHIKV pre-immunity across the older population.

**Table 2:** HIA and FRNT_90_ titers among the study participant subgroups (*n* = 154)

Subgroup	Subdivisions	Mean HIA	Mean FRNT_90_ Thailand 1958	Mean FRNT_90_ Thailand 1975	Mean FRNT_90_ New Caledonia 2011	Mean FRNT_90_ Cambodia 2011
		(95% CI)	(95% CI)	(95% CI)	(95% CI)	(95% CI)
—	Total	378	1028	398	507	1033
	(*n* = 154)	(286–469)	(841–1216)	(311–486)	(413–601)	(847–1219)
1: Age	Born before 1975	320	1000	393	505	862
	(*n* = 74)	(199–442)	(726–1274)	(283–502)	(362–648)	(726–1274)
	Born after 1980	431	1054	404	509	**1191**
	(*n* = 80)	(294–567)	(791–1318)	(267–540.1)	(382–636)	(912–1471)
2: IgM status	IgM negative	192	701	285	348	537
	(*n* = 80)	(117–266)	(487–914)	(194–376)	(234–462)	(370–703)
	IgM positive	**579**	1383	520	679	**1570**
	(*n* = 74)	(416–741)	(1083–1682)	(371–670)	(534–824)	(1269–1871)
3: Age and IgM status	Born before 1975	183	838	366	437	477
	IgM negative (*n* = 40)	(127–239)	(534–1143)	(234–498)	(250–624)	(306–648)
	Born before 1975	**482**	1190	424	584	**1316**
	IgM positive (*n* = 34)	(230–733)	(703–1677)	(236–611)	(356–812)	(863–1769)
	Born after 1980	200	563	204	259	596
	IgM negative (*n* = 40)	(59–341)	(257–869)	(76–332)	(125–393)	(303–890)
	Born after 1980	**661**	1546	603	759	1786
	IgM positive (*n* = 40)	(444–879)	(1164–1928)	(372–834)	(569–950)	(1379–2194)

*CI* confidence interval, *HIA* hemagglutination inhibition assay, *FRNT* foci reduction neutralization test

Bold number means higher HIA or FRNT_90_ titer within the subgroup; underlined number means higher FRNT_90_ titer within the subdivision (*p*  < 0.05)

The neutralization activity (expressed as FRNT_90_ titers) of each serum sample was analyzed against the ECSA IOL strain that caused the Cambodian outbreak in 2012 and against the three Asian genotype strains of varying origins (Supplement Table S1). Of 154 serum samples, 126 (81.8%) sera showed positive FRNT_90_ titers against at least one of the four investigated viruses. The majority (121/126, 96%) of study participants demonstrated detectable cross-neutralization against all four tested CHIKV strains. We detected one individual born in 1934 with neutralizing antibodies detectable only against the Thailand strain from 1958 (FRNT_90_ = 821) and no neutralization (FRNT_90_titer below the cut-off of 20) against the Cambodian IOL strain or the other two Asian genotype strains. We also found one additional study participant born in 1963 with low neutralization activity directed against only the 1958 Thailand strain (FRNT_90_ = 113) and against the recent Asian genotype strain from New Caledonia (FRNT_90_ = 31). Further, there were three individuals with low-neutralizing antibody titers (all FRNT_90_ titers < 200) against the Cambodian IOL strain, the Asian genotype strains from New Caledonia, and the 1958 Thai strain, but not against the 1975 Thai strain. Two of these study participants were born before 1975, and the third was born in 2005.

Overall, 72/74 (97.3%) of all study participants who were infected in 2012 had detectable CHIKV-neutralizing antibodies in 2016 (Fig. 1). Two participants tested positive for CHIKV IgM in 2012 but were negative in HIA or FRNT in 2016. As the specificity of our in-house MAC-ELISA could be subject to cross-reactivity, these two results from 2012 were retrospectively considered as false positives.

As the number of people with neutralizing antibodies detected in 2016 differed considerably across age groups, we further compared the FRNT_90_ titers of study participants who showed neutralization against at least one of the four CHIKV strains(*n* = 126) and excluded all FRNT-negative individuals from this analysis. The comparison of the neutralization titers between the age groups (Fig. 2b) revealed a significant difference in the neutralizing antibody titers against the Cambodian IOL strain (*p* < 0.0001):the neutralizing antibodies titers were more than twofold greater in the younger population (geometric mean FRNT_90_ = 1219) compared to the older population (geometric mean FRNT_90_ = 511). Neutralization titers against the Asian genotype strains did not differ significantly across the two age groups. Neutralization patterns, however, differed significantly. We observed higher variations in the neutralization titers among the younger study participants. Despite the fact that the 2011 Cambodian IOL strain and the 1958 Thai strain belong to different genotypes, younger individuals showed similar neutralization activity against these two strains. The FRNT_90_ titers against both the Cambodian IOL strain and the 1958 Thai strain (geometric mean FRNT_90_ titers: 1219 and 1035, respectively) were more than twofold higher than those against the strains from New Caledonia and from the 1975 Thai strain (geometric mean FRNT_90_ titers: 475 and 308, respectively; *p* values from 0.002 to <0.0001; Fig. 2b). We termed this observed neutralization pattern of comparable neutralization activity against the 2011 Cambodian IOL strain and the 1958 Thai strain “neutralization cluster”. Since neutralizing antibodies against CHIKV mainly target the envelope proteins E1 and E2, we compared these sequences for all strains used for the FRNT (Supplement Table S3). We identified only minor differences in the amino acid sequences of the envelope glycoproteins: the E1 sequence homology averaged 97.5% (9–10 amino acid difference among a total of 439 positions) between the Cambodian IOL strain and all investigated Asian genotype strains, and the E2 sequence homology averaged 94.8% (18–20 amino acids differing among a total 420 positions).

The older population showed the highest neutralization activity against the 1958 Thailand strain (geometric mean FRNT_90_ = 644), which was twofold greater than the value of neutralization titers against the other two Asian genotype strains (1975 Thailand strain: geometric mean FRNT_90_ = 197, *p* = 0.0008; New Caledonia strain: geometric mean FRNT_90_ = 297; *p* = 0.0153; Fig. 2b).

### Subgroup 2: Influence of CHIKV infection in 2012 on the neutralization activity against various CHIKV strains

**Fig. 3:**
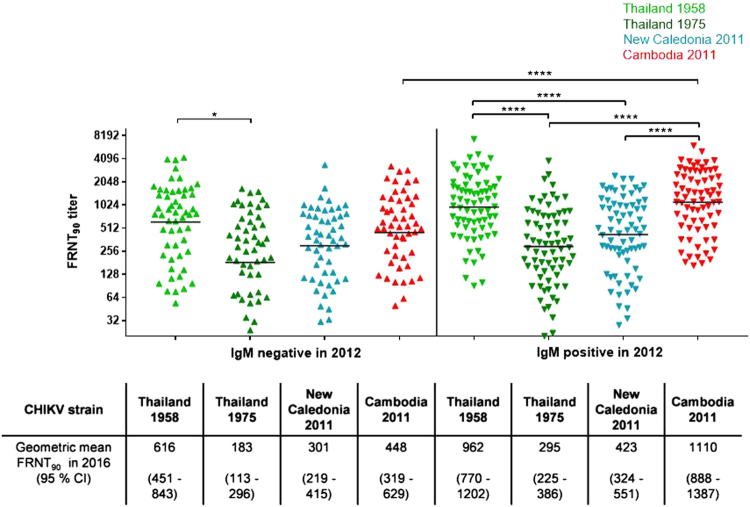
Subgroup 2: Influence of infection in 2012 on neutralizing antibody levels. Individual FRNT_90_ titers (with geometric mean) of study participants with neutralizing antibodies (*n* = 126), stratified by participants infected in 2012 (IgM positive, *n* = 54; down-pointing triangle) and participants not infected in 2012 (IgM negative, *n* = 72; up-pointing triangle). FRNT_90_ titers against the three Asian genotype strains from Thailand (TH 35: light green; TH 1455-75: dark green) and New Caledonia (NC-2011-568: blue) as well as the Cambodian ECSA IOL strain from 2011 (V1024306_KH11_PVH: red). Asterisks indicate statistically significant differences in mean FRNT_90_ titers between the distinct virus strains (*p < 0.05, ****p < 0.0001; one-way ANOVA, Tukey’s multiple comparison test)

As the age of the study group had an influence on the neutralization pattern across the four investigated CHIKV strains, we also analyzed the influence of a recent infection with the 2011 CHIKV IOL by comparing individuals who tested IgM positive in 2012 with those found to be IgM negative at the time. The HIA titers were nearly threefold in IgM-positive study participants compared to IgM-negative individuals, with mean HIA titers of 579 and 192 (*p* < 0.0001; Table 2). Moreover, the FRNT_90_ titers of study participants with neutralizing antibodies(*n* = 126) differed significantly only for the Cambodian IOL strain and the 1958 Thai strain (Fig. 3): the FRNT_90_ titers for the Cambodian IOL strain among IgM-positive individuals were more than twofold (geometric mean FRNT_90_ = 1110) greater than those among the IgM-negative participants (geometric mean FRNT_90_ = 448; *p* < 0.0001). Among the IgM-positive participants from 2012, the neutralization titer against the Cambodian IOL strain was also more than threefold greater than that against the Asian genotypes strain from Thailand in 1975 (geometric mean FRNT_90_ = 295; *p* < 0.0001) and twofold greater than that against the New Caledonian strain (geometric mean FRNT_90_ = 423; *p* < 0.0001) but was comparable to the neutralization titer against the Asian genotype strain isolated in Thailand in 1958 (geometric mean FRNT_90_ = 962). Therefore, the group of individuals infected with CHIKV in 2012 exhibited the neutralization cluster described above among people born after 1980, with significantly higher neutralization levels against the Cambodian IOL strain and the 1958 Thai strain.

### Subgroup 3: Influence of CHIKV infection in 2012 and age on the neutralization activity against various CHIKV strains

We compared the HIA titers between study participants infected during the 2012 outbreak with those of IgM-negative individuals, stratified by age group. Compared with the corresponding IgM-negative study participants, the IgM-positive study participants of both age groups (born before 1975 and after 1980) had elevated HIA titers due to their recent infection (Table 2). The difference, however, was only significant for the younger population, in which the HIA titers among participants who were IgM positive in 2012 were three times those of participants who were IgM negative in 2012 (mean HIA titers: 661 and 200; *p* = 0.0008).

**Fig. 4:**
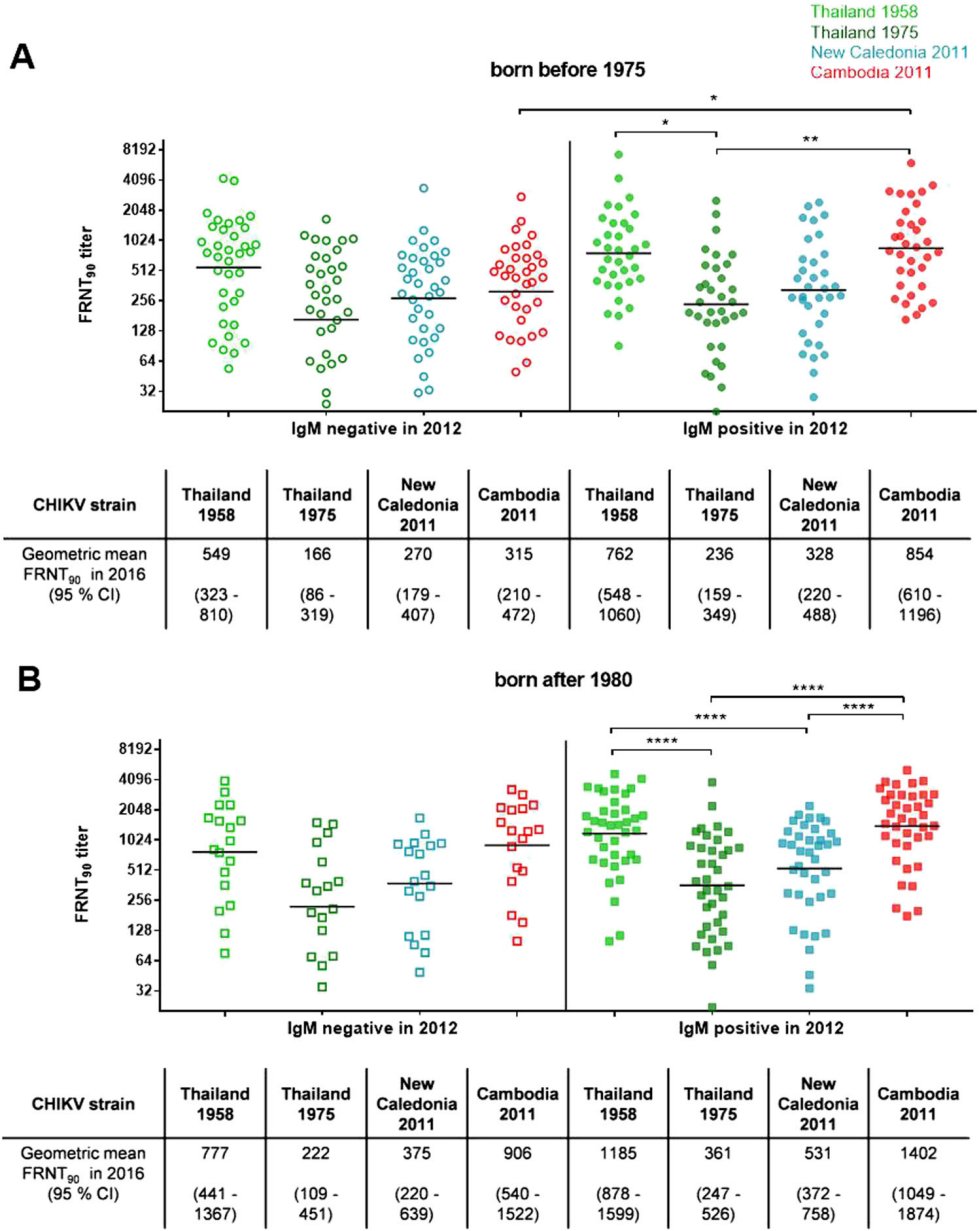
Subgroup 3: Influence of age and infection in 2012 on neutralizing antibody levels. Individual FRNT_90_ titers (with geometric mean) of study participants with neutralizing antibodies (*n* = 126). **a** FRNT_90_ titers of study participants born before 1975 divided by IgM status in 2012 (open symbols: negative; filled symbols: positive). **b** FRNT_90_ titers of study participants born after 1980 stratified by IgM status in 2012 (open symbols: negative; filled symbols: positive). FRNT_90_ titers against the three Asian genotype strains from Thailand (TH 35: light green; TH 1455-75: dark green) and New Caledonia (NC-2011-568: blue) as well as the Cambodian ECSA IOL strain from 2011 (V1024306_KH11_PVH: red). Asterisks indicate statistically significant differences in the mean FRNT_90_ titers between the various virus strains (*p < 0.05; **p < 0.01; ****p < 0.0001; one-way ANOVA, Tukey’s multiple comparison test)

A comparison of the neutralization titers among the study participants with detectable antibody levels (*n* = 126) again identified neutralization clusters across individuals who were IgM positive in 2012 (Fig. 4). The younger study participants infected in 2012 displayed neutralization titers against the Cambodian IOL strain (geometric mean FRNT_90_ = 1402) and the 1958 Thai strain (geometric mean FRNT_90_ = 1185) that were twofold greater than those against the strain from New Caledonia (geometric mean FRNT_90_ = 531) and the 1975 Thai strain (geometric mean FRNT_90_ = 361). Additionally, the individuals born before 1975 who were infected during the outbreak in 2012 had FRNT_90_ titers against the Cambodian IOL strain that were more than twofold greater than those found among the IgM-negative study participants from the same age group (geometric mean FRNT_90_ titers: 854 and 315; *p* = 0.0224). Such a difference among the study participants with neutralizing antibodies was not observed in the younger group, in which the FRNT_90_ titers against the Cambodian IOL strain in study participants infected during the 2012 outbreak and their non-infected counterparts were comparable (geometric mean FRNT_90_ titers: 1402 and 906, respectively). Neither the FRNT_90_ titers against the 1975 Thai strain nor those against the 2011 New Caledonian strain differed significantly between IgM-positive and IgM-negative study participants in both age groups. Individuals within the older age group who were infected with CHIKV in 2012 had significant higher neutralization titers against the Cambodian IOL strain than their uninfected counterparts. Additionally, these infected people showed twofold greater FRNT_90_ titers against the 1958 Thai strain (geometric mean FRNT_90_ = 762) compared to the other Thai strain isolated in 1975 (geometric mean FRNT_90_ = 236; *p* = 0.0359).

## Discussion

Our recent study on the CHIKV immunity in Cambodia after the outbreak in a rural community in 2012 found broad cross-neutralization between the ECSA IOL and the Asian genotype. Even 4 years after the outbreak, study participants infected in 2012 had significantly higher levels of neutralizing antibodies detected against the IOL strain and, surprisingly, against the Asian genotype strain isolated in Thailand in 1958. In contrast, neutralization activity against the two other tested Asian genotype strains (Thailand 1975 and New Caledonia 2011) was much lower and did not differ significantly between people infected in 2012 and the participants found to be IgM negative in 2012,considered as individuals who acquired their immunity at an unknown time point. We therefore propose the term neutralization cluster, dividing the four strains used in our analysis into two groups. We found significant differences in the neutralization patterns against the two closely related Thailand strains, both belonging to the Asian genotype. As the neutralizing antibodies mainly target the E1 and E2 glycoproteins on the surface of the chikungunya virions, we compared the amino acid sequences of the four CHIKV strains. This comparison revealed only three varying positions: E1-1 and E1-304, as well as E2-254. The two Thai strains that induced dissimilar neutralization titers differ in only one amino acid in each E1 and E2: a proline in position E1-304 and an isoleucine in position E2-254 in the 1958 Thai strain are replaced by a serine and a valine, respectively, in the 1975 Thai strain. At these positions, the 1958 Thai strain contained the same amino acids as the Cambodian IOL strain. This may explain why both strains had similar neutralization patterns, at least in participants born after 1980 (Table 3). The differing position in the E2 protein is part of a formerly described B cell epitope^38^. Within this epitope, Kam et al. showed that residue E2-525 was crucial for the neutralization potency, as a K525Q substitution changed the recognition of a neutralizing monoclonal antibody^38^. This area is also part of an acid-sensitive region that regulates the binding of neutralizing antibodies and conformational changes of the E2 protein^44^.

**Table 3:** Comparison of E1 and E2 amino acid sequences of the CHIKV strains used for neutralization assay

			Amino acid in the respective position			
Structural protein	Polyprotein position	Protein position	Thailand 1958	Thailand 1975	New Caledonia 2011	Cambodia 2011
E1	809	1	Met	Leu	Leu	Met
E1	1122	304	Pro	Ser	Ser	Pro
E2	579	254	Ile	Val	Val	Ile

Our study also supports the hypothesis raised by the 2012 field investigation that the Cambodian population born before 1975 acquired a cross-reactive CHIKV pre-immunity due to exposure in the 1960s to the Asian genotype of CHIKV,lasting over decades^26^. We observed a significantly lower proportion of naive individuals in the older population compared to the study participants born after 1980. This suggests that people born before 1975 were more exposed to CHIKV than the younger study participants until the 2012 outbreak. Long-lasting immunity was reported from an investigation in Thailand, where CHIKV antibodies were detected 19 years after a documented outbreak^30^. The antibody levels observed in the older population likely result from infection in the 1960s^24^, supporting the hypothesis of lifelong immunity against CHIKV. Long-lasting antibodies are reported after infection with other arboviruses, such as Dengue virus^45^ or West Nile virus^46^, and following yellow fever vaccination^47^. Another possibility is that the antibody response was boosted by subclinical infection(s) after the 1970s. There is, however, no evidence of CHIKV circulation after 1968 in Cambodia, partly due to a lack of documentation as a result of the civil war. There are reports of ongoing circulation in neighboring Thailand since the emergence of CHIKV in 1960^48^, with documented outbreaks in 1976, 1988, 1991, 1993, 1995, and, most recently,2008/2009, which was also caused by the IOL strain^49^. A national surveillance program for dengue-like diseases in Cambodia was implemented in 2000 and is carried out by the National Center for Parasitology, Entomology, and Malaria Control (CNM, Cambodian Ministry of Health) for syndromic surveillance and by IPC for virological surveillance. This program found no evidence of CHIKV circulation until its re-emergence in 2011^15^. This highly likely lifelong immunity should therefore further encourage the development of a vaccine against CHIKV. Another result of our study that supports vaccine development was the broad cross-neutralization that we observed across three different Asian genotype strains and the Cambodian IOL strain. The different CHIKV genotypes are antigenically extremely similar. Cross-reactivity was formerly reported between different CHIKV strains^50^ and even with other alphaviruses^51^ such as O’nyong-nyong virus^35, 52^. The ECSA genotype, especially the new IOL lineage, is highly divergent due to the adaptive mutations in the envelope proteins that increase the efficiency of transmission by *Ae. albopictus* mosquitoes^53^. The recent outbreaks in Asia were caused by the ECSA genotype, which replaced, in some countries, the formerly endemic Asian genotype strains^54^. Our findings showed that ECSA IOL infection induces broad immunity against both Asian and ECSA IOL strains, which might protect against future epidemics should the Asian genotype re-emerge in the region. This cross-protection strengthens the idea that a single vaccine can produce sufficient immunity against all CHIKV genotypes. As a result, developing a vaccine would likely be more accessible than for diseases caused by viruses with diverse serotypes such as dengue.

Our study has a few limitations. The sampling during the first outbreak investigation in 2012 was carried out using dry blood spots^25^, which allowed no additional or retrospective analysis by HIA or FRNT and therefore no comparison of the antibody titers between 2012 and 2016. Additionally, little is known regarding the circulation of CHIKV in Cambodia in general. Due to the Khmer Rouge regime (1975–1979) and the civil war that followed (until 1991), there is a prolonged information gap compared to other Asian countries such as Thailand, where continuous surveillance data are available since the 1960s, showing intermittent circulation of CHIKV^49^. Another limitation is that the samples used for our study were collected 4 years after the re-emergence of CHIKV in Cambodia. Additionally, the 2012 serosurvey of village residents was performed on a single day for the purpose of an outbreak investigation, possibly leading to missed IgM-positive individuals^25^. Furthermore, samples collected in 2012 were not tested for CHIKV IgG antibodies but only for IgM, allowing the possibility that some study participants might have been exposed to CHIKV between this investigation and the sampling done in 2016, although careful surveillance found little circulation of CHIKV after the initial 2011 emergence (Dussart P, 2017, unpublished. data). As the 2012 investigation analyzed only IgM and not IgG antibodies, we were unable to determine the time of CHIKV exposure in persons uninfected in 2012 who had neutralizing antibodies in 2016. Moreover, there might be an underestimation of CHIKV infections during the 2012 serosurvey, partially explained by the lack of sensitivity of the MAC-ELISA performed on dry blood spots or associated with self-reported symptoms by participants that might be less reliable. We, however, observed a significant difference between the older and younger study participants. Among the older population, the FRNT_90_ titers against the Cambodian IOL strain were significantly higher among people infected during the 2012 village outbreak compared to individuals who were not infected. In contrast, the FRNT_90_ titers did not differ significantly between IgM-negative and IgM-positive younger individuals. This is perhaps because study participants born after 1980 and uninfected in 2012 acquired the immunity that we observed in 2016 through infection with a CHIKV ECSA strain after 2012, whereas the immunity in the older people (born before 1975) uninfected in 2012 maybe due to an infection before 2012 with an Asian CHIKV genotype.

Overall, our immunological investigation of a chikungunya outbreak in a Cambodian rural community caused by an ECSA IOL strain found evidence of very long-lasting, likely lifelong immunity ranging across the CHIKV genotypes. Territories affected by the recent ECSA epidemicare likely to experience a silent inter-endemic phase in which the acquired population immunity prevents the re-emergence of CHIKV^55^. Nevertheless, experience from CHIKV surveillance in other countries shows that outbreaks are unpredictable, with irregular intervals. Efforts should be renewed toward the development of a chikungunya vaccine.

## Electronic supplementary material

Supplement Table S1

Supplement Table S2

Supplement Table S3
