# Supplementary material for: Broad and long-lasting immune protection against various Chikungunya genotypes demonstrated by participants in a cross-sectional study in a Cambodian rural community
Source: Emerg Microbes Infect. 2018 Feb 7;7:13. doi: 10.1038/s41426-017-0010-0 (PMC5837154; doi:10.1038/s41426-017-0010-0)
Supplement: Supplementary file 1 — Supplement Table S1 [file 41426_2017_10_MOESM1_ESM.docx]

# Supplementary information

**Supplementary Table S1.** CHIKV strains isolated from humans used for neutralization assay

| Strain | Country of Isolation, year | GenBank accession number | Genotype | Rational |
| --- | --- | --- | --- | --- |
| TH 35 | Thailand, 1958 | HM045810 | Asian | CHIKV strain representing the emergence of chikungunya in Southeast Asia in the 1950s-1960s.^43^ |
| TH 1455-75 | Thailand, 1975 | AF192898 | Asian | CHIKV strain used as a proxy for Cambodia, pre-Khmer Rouge regime (1975‑79) and civil war (until 1991).^1^ |
| NC-2011-568 | New Caledonia, 2011 | HE806461 | Asian | CHIKV strain circulating in early 2011 period in Southeast Asia and Western Pacific region.^44^ |
| V1024306_KH11_PVH | Cambodia, 2011 | JQ861253 | East Central South African (ECSA), Indian Ocean lineage (IOL) | CHIKV strain isolated during the re-emergence in Cambodia at the end of 2011.^15^ |
