# Supplementary material for: Broad and long-lasting immune protection against various Chikungunya genotypes demonstrated by participants in a cross-sectional study in a Cambodian rural community
Source: Emerg Microbes Infect. 2018 Feb 7;7:13. doi: 10.1038/s41426-017-0010-0 (PMC5837154; doi:10.1038/s41426-017-0010-0)
Supplement: Supplementary file 2 — Supplement Table S2 [file 41426_2017_10_MOESM2_ESM.docx]

# Supplementary information

**Supplement Table S2.** Spearman correlation between HIA test and FRNTs

| Spearman r value | HIA | FRNT_90_ Thailand 1958 | FRNT_90_ Thailand 1975 | FRNT_90_ New Caledonia 2011 |
| --- | --- | --- | --- | --- |
| FRNT_90_ Thailand 1958 | 0.829 |  |  |  |
| FRNT_90_ Thailand 1975 | 0.829 | 0.899 |  |  |
| FRNT_90_ New Caledonia 2011 | 0.862 | 0.923 | 0.918 |  |
| FRNT_90_ Cambodia 2011 | 0.887 | 0.907 | 0.855 | 0.903 |

Abbreviation: hemagglutination inhibition assay (HIA); foci reduction neutralization test (FRNT).
