# Supplementary material for: Broad and long-lasting immune protection against various Chikungunya genotypes demonstrated by participants in a cross-sectional study in a Cambodian rural community
Source: Emerg Microbes Infect. 2018 Feb 7;7:13. doi: 10.1038/s41426-017-0010-0 (PMC5837154; doi:10.1038/s41426-017-0010-0)
Supplement: Supplementary file 3 — Supplement Table S3 [file 41426_2017_10_MOESM3_ESM.docx]

# Supplementary information

**Supplement Table S3.** Percentage of amino acid identity of coding sequence of the E1 and E2 glycoprotein

| **E1 glycoprotein** All four strains: 428/439 identical positions, **97.5% identity** | | | |
| --- | --- | --- | --- |
| **Strain** | **Thailand 1958** | **Thailand 1975** | **New Caledonia 2011** |
| **Thailand 1975** | 438/439; 99.8% |  |  |
| **New Caledonia 2011** | 435/439; 99.1% | 436/439; 99.3% |  |
| **Cambodia 2011** | 430/439; 97.9% | 429/439; 97.7% | 429/439; 97.7% |
|  | | | |
| **E2 glycoprotein** All four strains: 398/420 identical positions, **94.8% identity** | | | |
| **Strain** | **Thailand 1958** | **Thailand 1975** | **New Caledonia 2011** |
| **Thailand 1975** | 418/420; 99.5% |  |  |
| **New Caledonia 2011** | 414/420; 98.6% | 419/420; 99.8% |  |
| **Cambodia 2011** | 402/420; 95.7% | 402/420; 95.7% | 400/420; 95.2% |
